# Metabolomics in the Development and Progression of Dementia: A Systematic Review

**DOI:** 10.3389/fnins.2019.00343

**Published:** 2019-04-12

**Authors:** Yanfeng Jiang, Zhen Zhu, Jie Shi, Yanpeng An, Kexun Zhang, Yingzhe Wang, Shuyuan Li, Li Jin, Weimin Ye, Mei Cui, Xingdong Chen

**Affiliations:** ^1^State Key Laboratory of Genetic Engineering and Collaborative Innovation Center for Genetics and Development, School of Life Sciences, Fudan University, Shanghai, China; ^2^Fudan University Taizhou Institute of Health Sciences, Taizhou, China; ^3^Key Laboratory of Public Health Safety of Ministry of Education, Department of Epidemiology, School of Public Health, Fudan University, Shanghai, China; ^4^Institute of Neurology, Huashan Hospital, Fudan University, Shanghai, China; ^5^State Key Laboratory of Genetic Engineering, Metabonomics and Systems Biology Laboratory, School of Life Sciences, Fudan University, Shanghai, China; ^6^International Peace Maternity and Child Health Hospital, School of Medicine, Shanghai Jiao Tong University, Shanghai, China; ^7^Human Phenome Institute, Fudan University, Shanghai, China; ^8^Department of Medical Epidemiology and Biostatistics, Karolinska Institutet, Stockholm, Sweden

**Keywords:** metabolomics, lipidomics, metabolites, dementia, Alzheimer's disease, mild cognitive impairment

## Abstract

Dementia has become a major global public health challenge with a heavy economic burden. It is urgently necessary to understand dementia pathogenesis and to identify biomarkers predicting risk of dementia in the preclinical stage for prevention, monitoring, and treatment. Metabolomics provides a novel approach for the identification of biomarkers of dementia. This systematic review aimed to examine and summarize recent retrospective cohort human studies assessing circulating metabolite markers, detected using high-throughput metabolomics, in the context of disease progression to dementia, including incident mild cognitive impairment, all-cause dementia, and cognitive decline. We systematically searched the PubMed, Embase, and Cochrane databases for retrospective cohort human studies assessing associations between blood (plasma or serum) metabolomics profile and cognitive decline and risk of dementia from inception through October 15, 2018. We identified 16 studies reporting circulating metabolites and risk of dementia, and six regarding cognitive performance change. Concentrations of several blood metabolites, including lipids (higher phosphatidylcholines, sphingomyelins, and lysophophatidylcholine, and lower docosahexaenoic acid and high-density lipoprotein subfractions), amino acids (lower branched-chain amino acids, creatinine, and taurine, and higher glutamate, glutamine, and anthranilic acid), and steroids were associated with cognitive decline and the incidence or progression of dementia. Circulating metabolites appear to be associated with the risk of dementia. Metabolomics could be a promising tool in dementia biomarker discovery. However, standardization and consensus guidelines for study design and analytical techniques require future development.

## Introduction

Dementia has become a major global public health challenge, and brings a heavy economic burden to the patients, their families, and society. More than 46 million people worldwide suffered from dementia in 2015; this figure is predicted to increase to 131.5 million by 2050 (Prince et al., 2015). The annual global cost for dementia care was estimated to be one trillion dollars by 2018, which is more than 1.1% of the global gross domestic product (Wimo et al., 2017). There is currently no effective disease-modifying treatment for dementia. Because of the long preclinical phase, in which there are early pathogenic changes but no cognitive impairment, prevention of dementia through management of modifiable risk factors is likely a better therapeutic strategy than developing a cure (Livingston et al., 2017). Therefore, there is an urgent need to understand the pathophysiological process of dementia and to identify biomarkers predicting risk of dementia in the preclinical stage, which could facilitate disease prevention, monitoring, and treatment (Livingston et al., 2017).

The definition of accurate preclinical biomarkers is essential to the early recognition of dementia. Clinical methods currently in use for the detection of dementia include evaluation of structural imaging (CT or MRI) for brain atrophy, functional MRI (fMRI) for brain connectivity, positron emission tomography (PET) for amyloid deposits, proton magnetic resonance spectroscopy (^1^H-MRS) for brain metabolites, and levels of cerebrospinal fluid (CSF) β-amyloid (Aβ) and tau (Ahmed et al., 2014). However, these means are limited because they are costly, partly invasive, time-consuming, and are not suitable for community-based population screening for early detection. Extensive deposition of Aβ was observed when MRI changes occurred, and targeting of Aβ failed to improve cognition in patients with Alzheimer's disease (AD) in phase III trials of anti-amyloid antibody treatments (Mullard, 2016), which highlighted the importance of early identification of dementia and discovery of additional biomarkers. Determination of reliable blood- or urine-based biomarkers could be an attractive development (Thambisetty and Lovestone, 2010; Ahmed et al., 2014; Fiandaca et al., 2014; Henriksen et al., 2014).

Metabolomics, an “omics” science, is based on high-throughput identification and quantification of small molecule metabolites in cells, tissues, and biofluids, and is a powerful tool for mapping global biochemical changes and discovery of new biomarkers in disease (German et al., 2005). Recently, metabolomics in animal models and human participants (in postmortem brain tissue, CSF, or blood) have attracted interest for the understanding of pathophysiology and identification of biomarkers of dementia (Kaddurah-Daouk et al., 2008; Xu et al., 2012; Fiandaca et al., 2014). Many metabolic changes in the brain or CSF could be reflected in blood profiles; metabolites in blood (serum or plasma) are therefore promising candidates for biomarkers of dementia (Fiandaca et al., 2014; Varma et al., 2018). Using proton (^1^H) nuclear magnetic resonance (NMR) or mass spectrometry (MS) metabolomics platforms, blood concentrations of many small molecule metabolites and lipids were found to be altered in patients with dementia relative to healthy controls (Xu et al., 2012; Wang et al., 2014; Proitsi et al., 2015; Li et al., 2016). However, these studies primarily aimed at differentiating patients with cognitive impairment from controls. This kind of cross-sectional design ignores the long preclinical phase of dementia that may be presented as a reverse causation, since the progression of dementia and many chronic complications associated with dementia could alter metabolite concentrations in peripheral blood (Thambisetty and Lovestone, 2010). Longitudinal studies allow direct assessment of pathobiology during transition periods, and identification of biomarkers for prediction of dementia. Several studies have fueled speculation of the discovery of circulating metabolites that influence cognition and the future development of dementia (Mapstone et al., 2014; Casanova et al., 2016; Bressler et al., 2017; Chouraki et al., 2017; Li et al., 2017; van der Lee et al., 2018; Varma et al., 2018). Identifying serum metabolic biomarkers of dementia in the preclinical stage would be a significant step forward, as such non-invasive blood-based biomarkers would reflect the disease-related pathophysiological process, and could be easily performed to identify individuals at risk, thus prevention and treatments could be initiated (Mielke et al., 2010; Oresic et al., 2011). However, no systematic review or meta-analysis of the use of metabolomics in prospective studies on dementia progression has been published.

Given the heterogeneity in study participants, samples (serum or plasma), and metabolite profiling analytical platforms, the aim of this systematic review is to examine and summarize recent human cohort studies assessing circulating metabolite markers, detected using high-throughput metabolomics, in the context of disease progression to dementia, including incident mild cognitive impairment (MCI), all-cause dementia, and cognitive decline (i.e., decline in neuropsychological test scores).

## Methods

### Data Sources and Search Strategy

The systematic search followed the *Cochrane Handbook of Systematic Reviews* (https://training.cochrane.org/handbook) and used the MOOSE (Meta-analysis Of Observational Studies in Epidemiology) checklist (Stroup et al., 2000). The review protocol has been registered in the PROSPERO International prospective register of systematic reviews (https://www.crd.york.ac.uk/PROSPERO/display_record.php?RecordID=111004). A comprehensive search of published literature in English was conducted in three databases: PubMed, Embase, and the Cochrane Library, from inception through October 15, 2018. The complete search strategy is listed in Supplementary Table 1. We also manually searched references of relevant articles and reviews during screening.

### Study Selection

All titles and abstracts were screened by two independent reviewers (Y.F.J. and Z.Z.); disagreements between them were resolved by full-text review and consensus. Studies were considered eligible for inclusion based on the following criteria: (1) cohort studies or other original studies in humans (e.g., cohort studies, case-cohort studies, nested case-control studies, or clinical trials); (2) metabolites in blood (plasma or serum) detected by high-throughput MS or NMR analytical platforms; (3) report of incident cognitive impairment (MCI or all-cause dementia) or cognitive decline (i.e., decline in neuropsychological test scores). Metabolomics studies in CSF, saliva, and urine samples and those using MRS for the quantification of brain metabolites were excluded. We also excluded cross-sectional studies, studies in unrelated patients, non-original studies (e.g., case reports, editorials, letter to editors, commentaries, expert opinions, and any type of reviews), experimental studies in animals, and duplicate publication of the same data.

### Data Extraction

Study characteristics (Table 1), including first author, year of publication and journal, study population and location, participant characteristics, sample size, duration of follow-up, biospecimens (plasma or serum), analytical technique and metabolite targets, outcome and ascertainment, number of observed events, statistical method, covariates included in the fully adjusted model, and a summary of key findings (analyzed metabolites and effect estimates), were extracted from each included study independently by two investigators (Y.F.J. and Z.Z.), and cross-checked by a third investigator (J.S.).

**Table 1:** Characteristics of studies investigating longitudinal associations between metabolites and dementia risk.

**References**	**Study population (country)**	**Study design**	***N* (% men), mean age (yrs), follow-up** **time (yrs)**	**Biospecimen**	**Baseline cognition status of participants**	**Outcome (*N*); ascertainment**
Mielke et al., 2010	WHAS II (USA)	Cohort, population-based	100 (0), 74.0, 9.0	Non-fasting serum	MMSE score ≥24 and none was impairment	Cognitive impairment on psychomotor speed (TMT-A, 24), executive function (TMT-B, 34), verbal immediate memory (HVLT-immediate recall, 27) and delayed (HVLT-delayed recall, 23) memory; first performance at or below the tenth percentile of each age- and education-matched cognitive test
Oresic et al., 2011	PredictAD project (Finland)	Patient cohort (internal cross-validation)	226 (37.6), 71.0, 2.3 (mean)	Fasting serum in most participants	MCI	Converters from MCI to AD (52); MCI, AD, and dementia were diagnosed using the criteria of proposed by MCADRC, NINCDS-ADRDA, and DSM-IV, repectively
Mapstone et al., 2014	Rochester/Orange County Aging Study (USA)	Case-control, longitudinal, population-based (internal cross-validation)	147 (37.4), 80.2, 2.1 (mean)	Fasting plasma	Non-impaired memory	Converters from non-impaired memory to aMCI/AD (74); aMCI and AD were classified as met the criteria of amnestic subtype of MCI, and NINCDS-ADRDA, repectively
Mousavi et al., 2014	Betula study (Sweden)	Case-control, longitudinal, population-based	93 (32.0), 65.6, 5.0	Non-fasting serum	Cognitively normal	Dementia (31); dementia, AD, and vascular dementia were diagnosed using the DSM-IV, NINCDS-ADRDA, Gorelick's criteria, repectively
Graham et al., 2015	Belfast City Hospital memory clinic patients (UK)	Case-control, longitudinal (internal cross-validation)	72 (45.8), 77.9, 2.0	Fasting plasma	MCI	Converters from MCI to AD (19); MCI and AD were classified as met the criteria of an international working group on MCI, and NINCDS-ADRDA, respectively
Casanova et al., 2016	BLSA (USA) and AGES-RS (Iceland)	Case-control, longitudinal, population-based (internal cross-validation)	BLSA: 192 (51.0), 77.2, 4.3 (mean); AGES-RS:200 (45.5), 78.2, 5.2 (mean)	Fasting serum	Cognitively normal	AD (93 in BLSA and 100 in AGES-RS); BLSA: DSM-III-R and the NINCDS-ADRDA criteria, for dementia and AD, respectively; AGES-RS: consensus made by a panel that includes a geriatrician, neurologist, neuropsychologist, and neuroradiologist based on a 3-step procedure, including cognitve test (MMSE, DSST), neurologic examination, and medical history and social, cognitive, and daily functioning relevant to the diagnosis
Simpson et al., 2016	BLSA-NI (USA)	Cohort, population-based	107 (61.0), 72.9, 7.0 (median)	Fasting plasma	Non-dementia	Cognitive decline; CVLT, TMT-A, TMT-B, MMSE, BVRT, CRT, and CLF were used for verbal memory, processing speed, executive function, global cognitive function, visual memory, visuo-spatial ability, and language fluencies, repectively
Abdullah et al., 2017	ADAPT (USA)	Case-control, longitudinal	195 (50.3), 78.0, 3.0	Non-fasting serum	Cognitively normal	MCI (15) and AD (8); Petersen criteria and NINCDS-ADRDA were used to diagnose MCI and AD, repectively
Bressler et al., 2017	ARIC-NS (USA)	Cohort, population-based	6-year cognitive change: 1,035 (33.0), 55.0, 6.0; Incident dementia: 1,534 (36.4), 53.4, 17.1 (median)	Fasting serum	NR	6-year cognitive change, difference between the cognitve test scores obtained at baseline and those at follow-up, including DWRT, DSST, and WFT; Dementia (141), hospital records
Chouraki et al., 2017	Framingham offspring Cohort (USA)	Cohort, population-based	2,067 (47.6), 55.9, 15.8 (mean)	Fasting plasma	Dementia-free	Dementia (93, including 68 AD); DSM-IV, and NINCDS-ADRDA for dementia and AD, respectively
Li et al., 2017	ARIC-NS (USA)	Cohort, population-based	221 (33.5), 71.3, 7.3 (median)	Fasting plasma	Cognitively normal	MCI (77), dementia (18) and cognitive score change; criterias of NIA-AA and DSM-V were used to classify MCI and dementia, repectively; cognitive test score changes in MMSE, DWRT, DSS, WFT, and a composite global score based on the above tests.
Toledo et al., 2017	Discovery: ADNI-1 (USA) Validation: ERF (the Netherland) RS (the Netherland)	Discovery: Patient cohort Validation: Cohort, population-based (external validation)	ADNI: 734 (42.4), 75.1, 3 (median) ERF: 905 (43.7), 48.0, NR RS: 2752 (41.8), 74.2, 9.7 (median)	Fasting serum	ADNI: 199 cognitivly normal, 358 MCI, and 175 AD ERF and RS: cognitivly normal	Converters from MCI to AD (NR); MCI and AD were diagnosed using criteria for aMCI and NINDS-ADRDA, respectively Cognitive change: ADAS-Cog13 in ADNI, general cognitive ability (g-factor) in ERS and RS based cognitive tests.
Dorninger et al., 2018	VITA (Austria)	Case-control, longitudinal, population-based	174 (36.7), 75.8, 7.5	Fasting plasma	Non-dementia	AD (22); DSM-IV and NINCDS-ADRDA were used to classify dementia and AD, respectively
Tynkkynen et al., 2018	Discovery: Finrisk 97 (Finland) DILGOM (Finland) WH II (UK) EGCUT (Estonia) Validation: Health 2000 (Finland) FHS (USA) RS (the Netherland) ERF (the Netherland)	Cohort, population-based (external validation)	A total of 22623; Finrisk 97: 4580 (50.4), 55.1, 10.0 (median) DILGOM: 3399 (47.0), 57.5, 7.9 (median) WH II: 4612 (73.5), 55.9, 17.0 (median) EGCUT: 2570 (41.1), 59.6, 7.5 (median) Health 2000: 1166 (42.9), 56.7, 10.0 (median) FHS: 2171 (47.4), 56.7, 17.9 (median) RS: 2759 (42.4), 75.0, 9.4 (median) ERF: 1366 (42.5), 49.2, 11.6 (median)	Fasting serum in six cohorts (except for Finrisk 97 fast for 4 h); Fasting plasma in FHS	Free of dementia	Dementia (995) and AD (745); based on continuous follow-up health records in the Finrisk97, ERF, WH II, EGCUT, Health 2000, and DILGOM; RS, based on the general practitioner records and cognitive screening met for DSM-III-R; FHS, based on cognitive test met for DSM IV (dementia) and NINCDS-ADRDA (AD).
van der Lee et al., 2018	Cognition analysis: Discovery: RS (the Netherland) ERF (the Netherland) Replication: WH II (UK) NTR (the Netherland) FHS (USA) SHIP (Germany) Dementia analysis: RS (the Netherland) ERF (the Netherland) WH II (UK) VUMC ADC (the Netherland) AgeCoDe (Germany) EGCUT (Estonia) Finrisk 97 (Finland) DILGOM (Finland)	Cohort, population-based (external validation)	Cognition analysis: Discovery: 5188 in total (2683 (43.1), 48.9 in ERF, 2505 (41.8), 74.2 in RS); Replication: 7,025 in total (4235 (73.8), 55.8 in WH II, 338 (37.6), 40.7 in NTR, 944 (43.6), 50.1 in SHIP, 1508 (54.9), 55.7 in FHS); Dementia analysis: 27,000 in total, 1532 in ERF, 11.3 (mean); 2010 in ERF 7.6 (mean); 4612 in WH II, 16.7 (mean); 2356 in FHS, 15.7 (mean); 1303 (54.9), 64.1 in VUMC ADC, NR; 7517 (45.3), 48.8 in Finrisk 97, 9.7 (mean); 4788 (44.2), 52.3 in DILGOM, 7.7 (mean); 2572 (41.1), 59.1 in EGCUT, 7.0 (mean); 310 (31.6), 84.1, in AgeCoDe, 4.5 (mean)	Fasting plasma (except for AUMC ADC and Finrisk 97)	Non-dementia for incident dementia analysis	Dementia (1990) and AD (1356); General cognitive ability score weres selcet as the first PCA component based on cognitive tests, including at least three cognitive domains. Dementia and AD: based on continuous follow-up health records in the ERF, WHII, EGCUT, Finrisk 97, and DILGOM; RS, based on the general practitioner records and cognitive screening met for DSM-III-R; FHS, based on cognitive test met for DSM IV (dementia) and NINCDS-ADRDA (AD); AgeCoDe, based on health records and cognitive tese met for DSM IV.
Varma et al., 2018	BLSA (USA) ADNI (USA)	BLSA: Cohort, population-based ADNI: Patient cohort,	BLSA: 207 (48.3), 78.7, 4.3 (mean) ADNI: 767 (57.4), 75.2, 3.0 (mean)	Fasting serum	Cognitively normal in BLSA; MCI in ADNI	Incident AD (92) and cognitive performance change in BLSA, converters from MCI to AD in ADNI (185); DSM-III-R and NINCDS-ADRDA was used to indentify dementia and AD, respectively; MCI was diagnosed using Petersen criteria. Cognitive performence was based on neuropsychological tests: CVLT for memory, TMT-A and Digit Forward for attention, TMT-B and Digit backward for executive function, Letter and Semantic Fluency for language, and CDT and CRT for visuo-spatial ability.

*ADAPT, Alzheimer's Disease Anti-inflammatory Prevention Trial; ADAS-Cog13, Alzheimer's Disease Assessment Scale–Cognition, lower levels indicate better cognition; ADNI, Alzheimer's Disease Neuroimaging Initiative; AgeCoDe, German Study on Aging, Cognition, and Dementia; AGES-RS, Age, Gene/Environment Susceptibility-Reykjavik Study; ARIC-NS, Atherosclerosis Risk in Communities-Neurocognitive Study; BLSA-(NI), Baltimore Longitudinal Study of Aging-(neuroimaging substudy); BVRT, Benton Visual Retention Test; CDT, Clock Drawing Test; CLF, Category and Letter Fluency Test; CRT, Card Rotation Test; CVLT, California Verbal Learning Test; DILGOM, Dietary, Lifestyle and Genetic determinants of Obesity and Metabolic Syndrome Study; DSM, Diagnostic and Statistical Manual of Mental Disorders; DSST, Digit Symbol Substitution Test; DWRT, Delayed Word Recall Test; EGCUT, Estonian Biobank (Estonian Genome Center, University of Tartu); ERF, Erasmus Rucphen Family study; FHS, Framingham Heart Study; HVLT, Hopkins Verbal Learning Test; MCADRC, Mayo Clinic Alzheimer's Disease Research Center; MCI, mild cognitive impairment; MMSE, Mini-Mental State Examination; NINCDS-ADRDA, National Institute of Neurologic and Communicative Disorders and Stroke, and Alzheimer's Disease and Related Disorders Association criteria; NTR, Netherlands Twin Registry; NR, not reported; RS, Rotterdam Study; SHIP-Trend, Study of Health in Pomerania–Trend; TMT, Trail Making Test; VUMC ADC, VUMC Amsterdam Dementia Cohort; WFT, Word Fluency Test; WH II, Whitehall II study; WHAS II, Women's Health and Aging Study (WHAS) II; yrs, years*.

### Quality Assessment

The quality of the studies were assessed and scored independently by two researchers (Z.Z. and J.S.) by using a modified Newcastle-Ottawa Scale (Wells et al., 2014). Cohort studies were scored on the following criteria: (1) Selection, including representativeness of source population (exposed and non-exposed cohort), ascertainment of exposure and definition of controls (1 point per criterion); (2) Comparability−2 points if the analysis was adjusted for most potential confounders, and 1 point if the analysis was only adjusted for age and sex (or age and sex matched); (3) Outcome, including assessment of outcome and duration of follow-up (1 point per criterion). Since all studies involved participants with baseline metabolic profiling and cognitive follow-up status, we removed attrition from the criteria. Case-control studies were rated on the following criteria: (1) Selection, including adequate definition of cases, representativeness of cases, and selection and definition of controls (1 point per criterion); (2) Comparability, same as cohort studies; (3) Exposure, including assessment of exposure, and same method of ascertainment for cases and controls (1 point per criterion). Points were summed and ranged from 0 to 8; studies with summed scores ≤4 were considered of low quality, while summed scores 5–8 were considered of high quality. Details of these criteria and score of each study are presented in Supplementary Tables 2, 3. Disagreements between reviewers in quality assessment were resolved through consensus.

## Results

### Literature Retrieval

**Figure 1:**
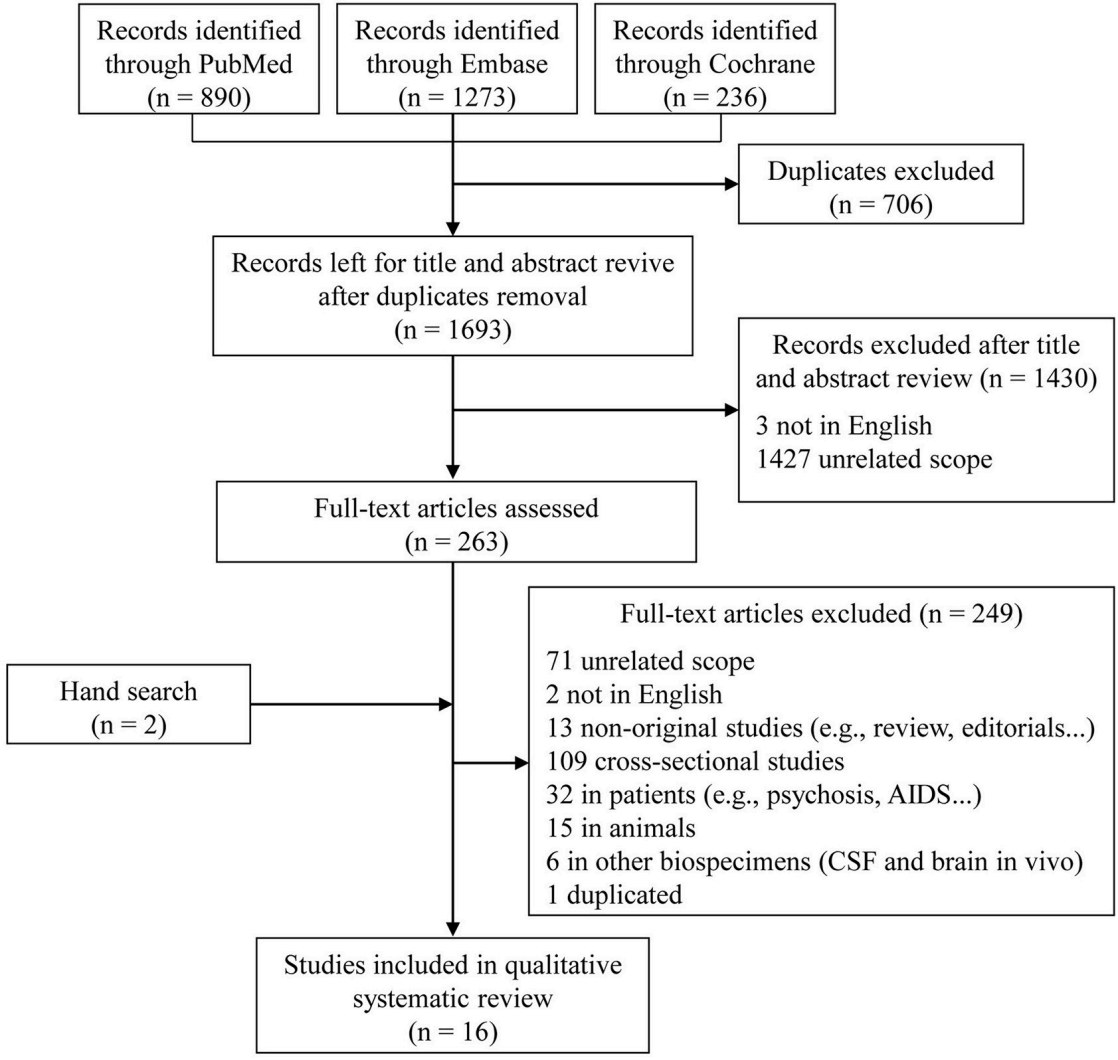
Flow diagram of literature search and study selection.

In the initial search, 1,693 unique studies were identified after removal of duplicates (Figure 1). Following the review of titles and abstracts, 263 articles were selected for careful full-text screening. Fourteen were eligible. Most of the 249 studies excluded were excluded because of unrelated scope, cross-sectional design, or they were conducted in patients or in animals, or were non-original studies. Six studies were conducted using other types of biological samples (i.e., CSF and brain *in vivo*). We also excluded a sub-analysis study (Fiandaca et al., 2015) using the same participants as a previous publication (Mapstone et al., 2014). In total, 16 full-text articles were eligible, with an additional two included after a hand search, for inclusion in this qualitative systematic review (Mielke et al., 2010; Oresic et al., 2011; Mapstone et al., 2014; Mousavi et al., 2014; Graham et al., 2015; Casanova et al., 2016; Simpson et al., 2016; Abdullah et al., 2017; Bressler et al., 2017; Chouraki et al., 2017; Li et al., 2017; Toledo et al., 2017; Dorninger et al., 2018; Tynkkynen et al., 2018; van der Lee et al., 2018; Varma et al., 2018).

### Study Characteristics

The characteristics of the 16 included studies are summarized in Table 1. All included studies were conducted in European or the US populations, and more than half (9/16) were published after 2016. The total number of participants was 61,345, ranging from 72 to 27,000, with mean age at baseline ranging from 48.0 to 84.1 years, and studies generally including more women than men (overall: 52.3% women).

Of the 16 longitudinal analyses, 10 studies used a retrospective cohort design (Mielke et al., 2010; Oresic et al., 2011; Simpson et al., 2016; Bressler et al., 2017; Chouraki et al., 2017; Li et al., 2017; Toledo et al., 2017; Tynkkynen et al., 2018; van der Lee et al., 2018; Varma et al., 2018), four were case-control designs embedded in population-based cohort studies (Mapstone et al., 2014; Mousavi et al., 2014; Casanova et al., 2016; Dorninger et al., 2018), and the other two analyses were based on an outpatient cohort (Graham et al., 2015) and a placebo-controlled primary prevention trial (Abdullah et al., 2017), respectively. Validations in external independent cohorts were conducted in only three of the studies (Table 1). Four studies included an internal replication analysis in randomly selected samples in the same population (Oresic et al., 2011; Mapstone et al., 2014; Graham et al., 2015; Casanova et al., 2016). Two articles aimed at replicating previously reported findings by Mapstone et al. (2014) using the same metabolite panels (Casanova et al., 2016; Li et al., 2017). Only three studies calculated and reported the sample size and expected statistical power (Mapstone et al., 2014; Casanova et al., 2016; Bressler et al., 2017), six articles acknowledged their small sample size as a limitation (Mielke et al., 2010; Oresic et al., 2011; Simpson et al., 2016; Abdullah et al., 2017; Toledo et al., 2017; Varma et al., 2018), and three analyses had a relatively large sample size or listed it as their strength (2,067, 22,623, and 27,000 respectively; Chouraki et al., 2017; Tynkkynen et al., 2018; van der Lee et al., 2018). The other four studies did not explicitly mention sample size (Mousavi et al., 2014; Graham et al., 2015; Li et al., 2017; Dorninger et al., 2018).

### Quality Assessment of Studies

The results of quality assessment of the included studies using a modified version of the Newcastle-Ottawa Scale are shown in Supplementary Tables 2, 3. Most studies (14/16) were considered high quality, with a total score ≥6. Low-quality studies with a total score of 4, mainly due to the representativeness of cases and controls, comparability, or not long enough duration of follow-up (<5 years), were present among the cohort and case-control studies.

### Biological Samples and Analytical Platform

Seven studies analyzed fasting plasma (except one non-fasting) and nine serum samples [five fasting and four non-fasting, while one study also used fasting plasma in the validation cohort (Tynkkynen et al., 2018)]. Variations of MS platforms were used in 13 studies, and three used both MS and NMR platforms (Table 2). More than half the studies used untargeted metabolomics analysis (*n* = 8). Targeted approaches were adopted in eight studies, and the BIOCRATES AbsoluteIDQ p180 platform was used in five of these analyses (Mapstone et al., 2014; Casanova et al., 2016; Li et al., 2017; Toledo et al., 2017; Varma et al., 2018).

**Table 2:** Results of analyses associating metabolites with dementia risk.

**References**	**Analytical platform, metabolite targets**	**Statistical analysis**	**Covariates in fully adjusted model**	Statistically significant and/or selected metabolites, adjusted HR (95% CI) for cognitive impairment per SD*	**Key findings**
Mielke et al., 2010	ESI/MS/MS, targeted (SM and ceramides)	Cox proportional hazards regression model	Age, glucose and BMI	**HVLT-delayed recall** (per tertile): Total SM: 1.88 (1.05–3.38) Ceramides: C16:0: 2.17 (1.17–3.84) C22:0: 2.12 (1.10–4.08) C24:0: 2.23 (1.23–4.05) Lactosyl C12:0: 2.26 (1.15–4.44) Stearoyl: 1.86 (1.05–3.27) Sulfatide: 2.06 (1.08–3.96)	High levels of serum SM could predict incident impairment in asymptomatic individuals, and be biomarkers of AD progression.
Oresic et al., 2011	UPLC-MS, untargeted (139 lipids: phospholipids, sphingolipids, and neutral lipids); GC × GC-TOFMS, untargeted (544 small polar metabolites: amino acids, free fatty acids, ketoacids, organic acids, sterols, and sugars)	Logistic regression model	Age, *APOE* ε4	Combination of three metabolits (PC (16:0/16:0), an unidentified carboxylic acid and 2,4-dihydroxybutanoic acid): OR, 8.0 (90% CI: 2.7–27.6) per unit increase	(1) Concentrations of ribose-5-phosphate was decreased, whereas 2,4-dihydroxybutanoic acid and lactic acid were upregulated in converters; (2) Combination of PC (16:0/16:0), an unidentified carboxylic acid and 2,4-dihydroxybutanoic acid predicted AD reasonably well, with AUC = 0.77 (90% CI: 0.65–0.87).
Mapstone et al., 2014	UPLC-ESI-QTOF-MS, untargeted (lipidomic profiling, 2,700 positive-mode features and 1,900 negative-mode features); SID-MRM-MS, targeted (184 small molecules and lipids)	LASSO penalty for putative metabolites selection, and logistic regression model for prediction analysis	Age, gender, education, and visit matched, and additional adjusted for *APOE* in prediction model	PC diacyl (aa) C36:6, PC aa C38:0, PC aa C38:6, PC aa C40:1, PC aa C40:2, PC aa C40:6, PC acyl-alkyl (ae) C40:6, lysoPC a C18:2, and AC (Propionyl AC (C3) and C16:1-OH); NR	(1) Baseline plasma levels of phosphatidylinositol, serotonin, phenylalanine, proline, lysine, PC, taurine and AC in converters were significant low; (2) A panel of lipids, comprising the 10 putative metabolites, could predict converters well, with AUC = 0.92 (95% CI: 0.87–0.98).
Mousavi et al., 2014	GC-TOF-MS, targeted, 208 metabolites	OPLS-DA	Age-, sex-,and education- matched	3,4-dihydroxybutanoic acid, docosapentaenoic acid, and uric acid; NR	Metabolites were different in serum in participants at the preclinical stage up to 5 years preceding dementia, despite that the cognitive performance were comparable with healthy controls.
Graham et al., 2015	UPLC-Q-TOF-MS, untargeted (6751 spectral features)	OPLS-DA	Age-matched	4-aminobutanal, GABA, L-ornithine, N1,N12-diacetlyspermine, N-acetylputrescine, spermine, creatine; NR	(1) Concentrations of 4-aminobutanal, GABA, L-ornithine were low, whereas N1,N12-diacetlyspermine, N-acetylputrescine, spermine, creatine were upgraded in converters relative to matched healthy controls; (2) Polyamine metabolism and L-arginine metabolism were disturbed in converters.
Casanova et al., 2016	FIA-MS/MS, targeted (AC, lipids, and hexoses); HPLC-MS/MS, targeted (amino acids and biogenic amines), 187 metabolites in total.	Logistic regression model, 4 machine learning methods (EN-RLR, RF, SVM, L1-RLR)	Age and sex matched	Propionylcarnitine, glutarylcarnitine, creatinine, methionine, ornithine, serine, taurine, threonine, glucose, PC aa C36:4, PC aa C38:4, PC ae C30:2, PC ae C42:5, and PC ae C44:4; NR	(1) A panel of 10 serum metabolites found by Mapstone et al. which could detect preclinical AD within 3 years, could not be replicated in the two cohorts; (2) A modest signal was found in one cohort (BLSA) with distinct metabolites associated with preclinical AD; however, the classification accuracies were not good, with AUC = 0.64 (95% CI: NR).
Simpson et al., 2016	UPLC-Q-TOF-MS, targeted (PC16:0/20:5, PC16:0/22:6, and PC18:0/22:6)	Generalized linear mixed model	Age, sex, education year, *APOE* ε4	None	(1) Baseline and changes in plasma PC concentrations were not associated with longitudinal changes in cognitive performance; (2) Dysregulation of peripheral PC metabolism may be a common feature of both AD and age-associated differences in cognition.
Abdullah et al., 2017	HPLC-MS, untargeted (lipidomics, including PC, PE, PI, lysoPC, and so on)	Cox proportional hazards regression model	Age, education, gender, creatinine, and treatment with statins or anti-hypertensive medications	Ratio of AA to DHA; NR	(1) High AA to DHA ratios were associated with the risk of developing MCI/AD within 3 years; (2) Combining the *APOE* genotypes, blood AA and DHA species and the Aβ42/Aβ40 ratio improves the accuracy for detecting preclinical MCI/AD; (3) An interaction between the ε4 status and high AA to DHA ratios was found with the risk of developing MCI/AD.
Bressler et al., 2017	GC-MS and LC-MS, untargeted (118 named and 86 unnamed metabolites)	Linear regression models were used for 6-year cognitive change analyses; Cox proportional hazards models were used for incident hospitalized dementia analyses	Age, gender, education, eGFR, DM, hypertension, BMI, LDL-C, current smoking, alcohol intake and *APOE* ε4	**6-year cognitive chang:** beta (SE) **(DWRT)** N-acetyl-1-methylhistidine: −0.656 (0.183), **(DSST)** Docosapentaenoate (n-6 DPA): 1.254 (0.320), **(DSST)** X-12844: 1.404 (0.391); **Incident dementia:** 4-androsten-3 beta, 17 beta-diol disulfate 1: 1.25 (1.11–1.40) pregnen-diol disulfate: 1.35 (1.17–1.56) 5 alpha-androstan-3 beta,17 beta-diol disulfate: 1.26 (1.12–1.42) X-11440: 1.37 (1.18–1.60) X-12851: 1.26 (1.12–1.43)	(1) Basline high levels of N-acetyl-1-methylhistidine and low levels of docosapentaenoate were significantly associated with greater 6-year change in DWRT and DSST scores; (2) Three sex steroid hormones (4- androsten-3 beta, 17 beta-diol disulfate 1, 5 alpha-androstan-3 beta, 17 beta-diol disulfate and pregnen-diol disulfate) were associated with an increased risk of dementia.
Chouraki et al., 2017	LC-MS, untargeted (54 amines and related metabolites, 59 organic acids and related metabolites, and 104 lipids)	Cox proportional hazards model	Age, sex, education, *APOE* ε4, total homocysteine, SBP, antihypertensive medication, DM, smoking, CVD, AF and left ventricular hypertrophy.	Dementia: Anthranilic acid: 1.38 (1.12–1.69) Glutamic acid: 1.33 (1.06–1.66) Taurine: 0.74 (0.59–0.92) Hypoxanthine: 0.74 (0.59–0.91); AD: Glutamic acid: 1.37 (1.04-1.79)	(1) Higher plasma anthranilic acid levels were associated with greater risk of dementia; (2) Higher plasma glutamic acid, lower taurine and lower hypoxanthine showed possible associations with greater dementia risk.
Li et al., 2017	HPLC-MS/MS and FIA-MS/MS, targeted (188 metabolites, including 40 AC, 21 amino acids, 21 biogenic amines, 15 sphingolipids, 90 glycerophospholipids, and 1 hexose)	Logistic regression models were used in prediction analysis for baseline and changes in 9 targeted metabolites and incident MCI and dementia; linear regression models were used for cognitive change analyses	Age, race, sex, *APOE*, education, DM, BMI, drinking, smoking, sports index, SBP, use of antihypertensive medications, CVD, HF, stroke, TC, HDL-C, and TG.	**MCI:** LysoPC a C18:2: 1.66 (1.04–2.64) **MCI and dementia:** LysoPC a C18:2: 1.52 (1.03–2.37) **Global cogintive function score change:** Propionyl-L-carnitine (C3): 0.11 (0.01-0.22)	(1) A panel of 10 serum metabolites found by Mapstone et al. which could detect preclinical AD within 3 years, was not predictive of MCI or dementia in ARIC-NS; (2) Higher concentrations of lysoPC a C18:2 was significantly associated with high risk of MCI or MCI and dementia; (3) Higher levels of propionyl-L-carnitine (C3) were significantly associated with slower decline in the global cognitive score; (4) Higher concentrations of 28 plasma amino acids, carnitines, phospholipids, and sphingomyelins were prospectively associated with MCI or dementia in African Americans.
Toledo et al., 2017	ADNI: UPLC-/MS/MS and FIA-MS/MS, targeted (186 metabolites, including amino acids, biogenic amines, cylcarnitines, SM, PCs, and lysoPC) ERF: ESI/MS/MS, targeted RS: NMR, untargeted	Cox proportional hazards model was used to evaluate the association of metabolite levels with progression from MCI to AD; Mixed effects model was used to evaluate the association of metabolites with change in ADAS-Cog13; Linear regression model was used to evaluate the association of metabolites and g-factor.	Discovery: Age, gender, *APOE* ε4, and education. Validation: Age, gender, lipid-lowering medication, and education. PC ae C44:4: 0.49 SM (OH) C14:1: 0.015 SM C16:0: 0.0009 SM C20:2: 0.11 α-AAA:−0.093 Valine:−0.0006 **Validation:** **g-factor:** beta (SE, NR) (ERF) PC ae C40:3:−0.231 (ERF) SM C20:2:−0.239 (RS) Valine: positive correlation (NR) **Incident AD:** (RS) Valine: negative correlation (NR)	**Discovery:** **Progression** **MCI to AD:** (95% CI, NR) PC ae C36:2: 1.056 PC ae C40:3: 5.98 PC ae C42:4: 1.96 PC ae C44:4: 5.89 SM (OH) C14:1: 1.08 SM C16:0: 1.004 SM C20:2: 1.9 **ADAS-Cog13 Change:** beta (SE, NR) C14:1: 1.39 PC ae C40:3: 0.38 PC ae C42:4: 0.15	(1) Six metabolites (PC ae C40:3, PC ae C42:4, PC ae C44:4, SM (OH) C14:1, SM C16:0, and SM C20:2) showed a positive association with risk of conversion from MCI to AD and cognitive score change; (2) Lower valine and α-AAA values were associated with faster cognitive decline; (3) Valine was strongly associated with a higher general cognitive ability, decrease in valine concentration was associated with risk of AD in the validation cohorts (RS).
Dorninger et al., 2018	UFLC-MS/MS, targeted (lysoPC, PlsCho, and lyso-PAF)	Baseline phospholipid difference in groups: *t*-test; Phospholipid changes: ANCOVA; Group difference in phospholipid changes: ANCOVA	Gender, group indicator, *APOE*, and intake of lipid-lowering drugs	total lysoPC, lysoPC 18:2, total PlsCho, and total lyso-PAF; NR	(1) Total lysoPC and total PlsCho levels were lower, whereas total lyso-PAF was higher at baseline in converters than those in healthy controls; (2) The levels of lysoPC, PlsCho, and lyso-PAF increase significantly during normal aging as well as in developed probable AD patients.
Tynkkynen et al., 2018	NMR analysis was used in all cohorts except for FHS (LC-MS), untargeted (228 metabolites, including lipids, fatty acids, amino acids, ketone bodies, and gluconeogenesis-relatedmetabolites)	Cox proportional hazards models	Age, sex, education grade, *APOE* ε4, SBP, hypertension treatment, DM, smoking, and any CVD.	**Demnetia:** Creatinine: 0.90 (0.83–0.97) Isoleucine: 0.87 (0.80–0.94) Leucine: 0.83 (0.76–0.90) Valine: 0.84 (0.78–0.91) L-HDL-CE-%: 1.11 (1.03–1.21) S-VLDL-C: 0.87 (0.81–0.94) XL-VLDL-C-%: 1.11 (1.02–1.20): 0.91 (0.84–0.99) **AD:** Isoleucine: 0.89 (0.81–0.98) Leucine: 0.88 (0.79–0.97) Valine: 0.87 (0.79–0.96) L-HDL-CE-%: 1.12 (1.01–1.23)	(1) Lower levels of the BCAA such as valine were associated with an increased risk of both all dementia and of AD; (2) Inverse associations of creatinine, total cholesterol in S-VLDL-C, and triglycerides to total lipids ratio in very large VLDL were found associated with incident dementia, but not with AD; (3) The concentration of L-HDL-CE-% was associated with an increased risk of AD.
van der Lee et al., 2018	ERF, RS, NTR, VUMC ADC, EGCUT, WHII, Finrisk 97, and DILGOM were used NMR platform, SHIP was used LC-MS/MS, FHS was used LC-MS, AgeCoDe was used GC-FID; untargeted (299 metabolites in discovery analysis, including lipids, fatty acids, amino acids, ketone bodies, and gluconeogenesis-relatedmetabolites)	Cox proportional hazards models (logistic regression was used in VUMC ADC)	Age, sex, BMI, lipid-lowering medication, and *APOE* ε4.	**Dementia:** Small particles -HDL-free cholesterol: 0.85 Medium particles -HDL-phospholipids: 0.90 DHA: 0.91 Glutamine: 1.08 Medium particles -HDL-cholesterol esters: 0.92 Medium particles-HDL-total cholesterol: 0.92 **AD:** Small particles -HDL-free cholesterol: 0.87 DHA: 0.89 Glutamine: 1.11	
Varma et al., 2018	FIA-MS/MS and HPLC-MS/MS, targeted (187 metabolites, including amino acids, biogenic amines, AC, lipids, and hexoses)	Machine-learning method (SVM and RF) was used to select potential brain metabolite signature of AD; Cox regression models were used to test blood metabolite associations with risk of conversion from normal cognition to incident AD in BLSA and risk of conversion from MCI to incident AD in ADNI.	Age and sex	**Incident AD** in BLSA: per log unit SM C16:0: 4.43 (1.70–11.52) SM C16:1: 3.46 (1.52–7.87) SM (OH) C14:1: 3.54 (1.37–9.12) SM C18:1: 2.26 (1.05–4.85) PC aa 38:4: 0.25 (0.10–0.63) PC ae C34:2: 3.06 (1.21–7.70) **Cognitive performence change** in BLSA: beta (SE) **Attention:** SM C18:1: −0.17 (0.07) PC aa C40:6: −0.12 (0.05) **Language:** arginine: −0.14 (0.07) lysoPC a C18:0: −0.15 (0.06) PC ae C40:1: −0.25 (0.12) SM C26:1: −0.53 (0.27) **Visuospatial ability:** arginine: 0.20 (0.10) spermidine: 1.22 (0.57) **Converters from MCI to AD in** ADNI: SM C18:1: 2.35 (1.27–4.36) PC aa 38:4: 2.38 (1.19–4.74)	Perturbations in sphingolipid metabolism may be integral to the evolution of AD neuropathology as well as to the eventual expression of AD symptoms in cognitively normal older individuals.

AA, arachidonic acid; AAA, aminoadipic acid; Aβ, β-amyloid; AC, acylcarnitines; ADAS-Cog13, Alzheimer's Disease Assessment Scale–Cognition, lower levels indicate better cognition; ADNI, Alzheimer's Disease Neuroimaging Initiative; AF, atrial fibrillation; AgeCoDe, German Study on Aging, Cognition, and Dementia; ANCOVA, analysis of covariance; APOE, apolipoprotein E; ARIC-NS, Atherosclerosis Risk in Communities-Neurocognitive Study; AUC, area under the curve; BCAA, branched-chain amino acids; BLSA-(NI), Baltimore Longitudinal Study of Aging-(neuroimaging substudy); BMI, body mass index; CI, confidence interval; CVD, cardiovascular disease; DHA, docosahexaenoic acid; DILGOM, Dietary, Lifestyle and Genetic determinants of Obesity and Metabolic Syndrome Study; DM, diabetes mellitus; DSST, Digit Symbol Substitution Test; DWRT, Delayed Word Recall Test; EGCUT, Estonian Biobank (Estonian Genome Center, University of Tartu); eGFR, estimated glomerular filtration rate; EN-RLR, elastic net regularized logistic regression; ERF, Erasmus Rucphen Family study; ESI/MS/MS, electrospray ionization triple stage quadruple tandem mass spectrometer; FHS, Framingham Heart Study; FIA-, flow injection analysis-; GC, gas chromatography; HDL-C, high density lipoprotein cholesterol; HF, heart failure; HPLC-, high-pressure liquid chromatography; HVLT, Hopkins Verbal Learning Test; L1-RLR, L1 regularized logistic regression; LASSO, least absolute shrinkage and selection operator; LC, liquid chromatography; LDL-C, low density lipoprotein cholesterol; L-HDL-CE-%, Cholesterol esters to total lipids ratio in large HDL; lyso-PAF, lyso-platelet activating factor; lysoPC, lysophophatidylcholine; MCI, mild cognitive impairment; MMSE, Mini-Mental State Examination; MS, mass spectrometry; NMR, nuclear magnetic resonance; NTR, Netherlands Twin Registry; NR, not reported; OPLS-DA, orthogonal projection to latent structures-discriminant analysis; PC, phosphatidylcholine; PCA, principal component analysis; PE, phosphatidylethanolamine; PI, phosphatidyl-inositol; PlsCho, choline plasmalogen; Q-TOF, quadrupole time-of-flight; RF, random forest; RS, Rotterdam Study; SBP, systolic blood pressure; SD, standard deviation; SHIP-Trend, Study of Health in Pomerania–Trend; SID-MRM-, stable isotope dilution–multiple reaction monitoring-; SM, sphingomyelins; S-VLDL-C, total cholesterol in small VLDL; SVM, support vector machines; TC, total cholesterol; TG, triglycerides; TMT, Trail Making Test; UFLC-, ultra-fast liquid chromatography; UPLC-, ultra-performance liquid chromatography coupled to-; VUMC ADC, VUMC Amsterdam Dementia Cohort; WFT, Word Fluency Test; WH II, Whitehall II study; WHAS II, Women's Health and Aging Study (WHAS) II; X-^*^, unnamed metabolites; XL-VLDL-C-%, total cholesterol to total lipids ratio in small VLDL; XL-VLDL-TG-%, triglycerides to total lipids ratio in very large VLDL; yrs, years; ^*^Except where stated otherwise.

### Outcome and Ascertainment

Six studies evaluated the relationship between baseline metabolites and cognitive performance changes, relying on a number of neuropsychological tests (Mielke et al., 2010; Simpson et al., 2016; Bressler et al., 2017; Li et al., 2017; Toledo et al., 2017; Varma et al., 2018). Conversion from MCI to AD/dementia was assessed in four of these studies (Oresic et al., 2011; Graham et al., 2015; Toledo et al., 2017; Varma et al., 2018), and 14 studies determined risk of MCI and/or all-cause dementia in cognitively normal populations, mainly nested in prospective population-based cohorts (Mapstone et al., 2014; Mousavi et al., 2014; Casanova et al., 2016; Abdullah et al., 2017; Bressler et al., 2017; Chouraki et al., 2017; Li et al., 2017; Dorninger et al., 2018; Tynkkynen et al., 2018; van der Lee et al., 2018; Varma et al., 2018). The neuropsychological test battery (Table 1) used in these studies included Mini-Mental State Examination (MMSE), California Verbal Learning Test (CVLT), Delayed Word Recall Test (DWRT), and Trail Making Test A/B (TMT-A/B). Various criteria were used to classify MCI based on cognitive tests, such as the one proposed by Petersen (Petersen et al., 1999). Assessment of dementia varied across studies from examination based on the Diagnostic and Statistical Manual of Mental Disorders (DSM, 4 III-R, 5 IV, and 1 V; Association, 1987, 1994, 2013) criteria only, examination with complementary medical records (Tynkkynen et al., 2018; van der Lee et al., 2018), or health records only (Bressler et al., 2017). All the classifications of AD were based on the National Institute of Neurological and Communication Disorders and Stroke-Alzheimer's Disease and Related Disorders Association (NINCDS-ADRDA) criteria (McKhann et al., 1984).

Participants in 13 studies were free of dementia at baseline (Mielke et al., 2010; Mapstone et al., 2014; Mousavi et al., 2014; Casanova et al., 2016; Simpson et al., 2016; Abdullah et al., 2017; Bressler et al., 2017; Chouraki et al., 2017; Li et al., 2017; Dorninger et al., 2018; Tynkkynen et al., 2018; van der Lee et al., 2018; Varma et al., 2018), while one of them contained a validation analysis in converters from MCI to AD (Varma et al., 2018). Individuals were diagnosed with MCI at baseline in three studies, which aimed to assess the baseline metabolites associated with progression to AD (Oresic et al., 2011; Graham et al., 2015; Toledo et al., 2017). The mean or median follow-up time after sample collection ranged from 2.0 to 17.9 years. The number of converters from MCI to AD, MCI, AD, and all-cause dementia in these studies ranged from 19 to 185, 15 to 77, 8 to 1356, and 18 to 1990, respectively (Table 1).

### Statistical Approaches

Cox proportional hazards regression models were applied in eight analyses to estimate the association between metabolites and the risk of incident all-cause dementia (Mielke et al., 2010; Abdullah et al., 2017; Bressler et al., 2017; Chouraki et al., 2017; Tynkkynen et al., 2018; van der Lee et al., 2018) or progression from MCI to AD (Toledo et al., 2017; Varma et al., 2018). This relationship was evaluated using logistic regression models in another four studies (Oresic et al., 2011; Mapstone et al., 2014; Casanova et al., 2016; Li et al., 2017). Linear models were typically used to assess the association of baseline circulating metabolites with changes in cognitive performance (Casanova et al., 2016; Bressler et al., 2017; Li et al., 2017; Toledo et al., 2017). Casanova et al. (2016) and Varma et al. (2018) additionally used several machine-learning methods, such as support vector machine (SVM) and random forest (RF), to evaluate the use of metabolites in discriminating between patients with cognitive impairment and healthy controls. Age and sex were adjusted or matched as covariates in all analyses except in Dorninger et al. (2018); some studies further adjusted for education, apolipoprotein E (*APOE* ε4), vascular risk factors (e.g., smoking, alcohol drinking, body mass index [BMI], hypertension or blood pressure, diabetes mellitus, and triglycerides), cardiovascular diseases, and lipid-lowering medication (Table 2).

### Metabolites Associated With Dementia Risk

Table 2 shows the results of analyses associating metabolites with dementia risk. Metabolites related to future cognitive decline were estimated individually in two studies (Mielke et al., 2010; Simpson et al., 2016) and nested in four other included studies (Bressler et al., 2017; Li et al., 2017; Toledo et al., 2017; Varma et al., 2018). Faster global cognitive decline risk was observed in participants with lower concentrations of acylcarnitine (Li et al., 2017), alpha-aminoadipic acid (α-AAA) (Li et al., 2017; Toledo et al., 2017), valine (Toledo et al., 2017), or higher concentrations of sphingomyelins (SM) and phosphatidylcholines (PC) (Toledo et al., 2017) at baseline. In terms of different cognitive domains, higher levels of SM, PC, and lysophophatidylcholine (lysoPC) were associated with deterioration in attention, language, and verbal memory (Mielke et al., 2010; Varma et al., 2018). Elevated ceramides were related to memory loss (Mielke et al., 2010), and amino acids such as arginine and histidine were associated with impairment in memory (Bressler et al., 2017), language, and visuospatial skills (Varma et al., 2018). However, baseline and changes in plasma PC concentrations were not associated with 7-year cognitive performance change in the study of Simpson et al. (2016).

The majority of the studies aimed at investigating lipids, biogenic amines, acylcarnitines (AC), and amino acids in the context of risk of incident MCI or dementia (Mielke et al., 2010; Oresic et al., 2011; Mapstone et al., 2014; Mousavi et al., 2014; Graham et al., 2015; Casanova et al., 2016; Abdullah et al., 2017; Bressler et al., 2017; Chouraki et al., 2017; Li et al., 2017; Toledo et al., 2017; Dorninger et al., 2018; Tynkkynen et al., 2018; van der Lee et al., 2018; Varma et al., 2018). Of the 16 studies, 10 assessed the association between lipid metabolites and dementia. SM, PC, and lysoPC were the most commonly identified potential biomarkers, and higher baseline levels of these lipids are likely an important indicator of a higher risk of MCI, and conversion from MCI to AD/dementia (Oresic et al., 2011; Li et al., 2017; Toledo et al., 2017; Varma et al., 2018). However, Mapstone et al. reported that PC was significantly lower in participants who later showed a conversion to MCI/AD compared to cognitively normal participants (Mapstone et al., 2014). Further, docosahexaenoic acid (DHA), a well-known long-chain omega-3 polyunsaturated fatty acid involved in brain development in early life, was associated with good cognitive performance and lower risk of dementia (Abdullah et al., 2017; van der Lee et al., 2018). Moreover, van der Lee et al. (2018) found that several high-density lipoprotein (HDL) subfractions were associated with higher cognitive ability and lower risk of dementia and AD. The triglycerides-to-total-lipids ratio and other similar ratios were associated with incident dementia in the study by Tynkkynen et al. (2018). However, we did not find any metabolites that were consistently identified in all studies. Only SM C16:0 and SM (OH) C14:1 were associated with conversion from MCI to AD in an Alzheimer's disease Neuroimaging Initiative (ADNI) study (Toledo et al., 2017) and incident AD in the Baltimore Longitudinal Study of Aging (BLSA) (Varma et al., 2018).

Some essential and non-essential amino acids were significantly associated with dementia. The study by Tynkkynen et al. (2018) demonstrated that increases in branched-chain amino acids (BCAAs, i.e., isoleucine, leucine, and valine), were associated with lower dementia risk, and similar results were found by Toledo et al. (2017), concerning valine. Glutamine and glutamate were associated with higher dementia risk in two studies (Chouraki et al., 2017; van der Lee et al., 2018). Creatinine (Tynkkynen et al., 2018) and taurine (Chouraki et al., 2017) were each associated with between 10 and 26% lower risk of dementia, whereas higher plasma anthranilic acid levels were associated with greater risk of dementia (Chouraki et al., 2017). In addition, baseline blood concentrations of gamma-aminobutyric acid (GABA), ornithine, and phenylalanine were lower (Mapstone et al., 2014; Graham et al., 2015), and dihydroxybutanoic acid levels were higher (Oresic et al., 2011; Mousavi et al., 2014) in dementia converters relative to healthy controls.

Three sex steroid hormones (4- androsten-3 beta, 17 beta-diol disulfate 1; 5 alpha-androstan-3 beta, 17 beta-diol disulfate; and pregnen-diol disulfate; Bressler et al., 2017), and high arachidonic acid (AA) to DHA ratios (Abdullah et al., 2017) were associated with an increased risk of dementia, whereas hypoxanthine (Chouraki et al., 2017) decreased dementia risk. Several other circulating metabolites were also involved in the physiological processes and progression of cognitive disorders (Table 2).

### Predictive Analysis

Seven studies assessed whether circulating metabolites significantly associated with dementia risk could be effectively used to distinguish or/and improve the power to predict MCI/dementia in a cognitively normal population (Oresic et al., 2011; Mapstone et al., 2014; Mousavi et al., 2014; Graham et al., 2015; Casanova et al., 2016; Abdullah et al., 2017; Li et al., 2017). Three studies reported a significant improvement in dementia prediction using combinations of metabolites relative to only traditional risk dementia factors (e.g., age, education, *APOE* ε4, Aβ, etc.; Oresic et al., 2011; Mapstone et al., 2014; Abdullah et al., 2017). However, only a small improvement in MCI/dementia prediction was observed in the study of Li et al, with only 0.03 increase in the C statistic (a measure of predictive ability; Li et al., 2017).

Using both untargeted and targeted MS-based platform analysis of 4600 features, Mapstone et al. (2014) identified a set of 10 lipids, after least absolute shrinkage and selection operator (LASSO) selection, that predicted MCI/AD reasonably well with an accuracy of >90% within a 3-year period. However, with the same metabolomics platform, Casanova et al. (2016) and Li et al. (2017) failed to replicate this 10-metabolite panel as predictor of incident MCI/dementia in three cohorts (i.e., BLSA, Age, Gene/Environment Susceptibility-Reykjavik Study [AGES-RS], and Atherosclerosis Risk in Communities-Neurocognitive Study [ARIC-NS]), with a C statistic of 0.642, 0.395, and 0.609, respectively.

## Discussion

Through our comprehensive systematic review, we identified 16 articles, including 22 analyses, which prospectively evaluated the association between circulating metabolites and risk of dementia, three of which reported good diagnostic or predictive performance. All the included studies were conducted in western populations, and most were population-based cohort studies. MS-based approaches were predominantly applied to profile metabolite features in these analyses. Generally, elevated concentrations of some metabolites such as SM, PC, and glutamate were associated with a higher risk of dementia, whereas other metabolites (e.g., DHA and BCAAs) were associated with a lower risk. Most of the studies focused on lipids in blood samples as promising biomarker candidates. However, the same set of metabolites was rarely found in different populations, making it difficult to draw consistent conclusions from these studies.

Variations in lipid, energy, amino acid, purine, and neurotransmitter metabolism, and oxidative stress were associated with dementia pathogenesis in many previous cross-sectional animal models and human patient metabolomics studies (Seshadri et al., 2002; Thambisetty and Lovestone, 2010; Xu et al., 2012; Ahmed et al., 2014; Jove et al., 2014; Trushina and Mielke, 2014; Gonzalez-Dominguez et al., 2017; Thambisetty, 2017; Yi et al., 2017). In this systematic review, we confirmed that alterations in lipids, amino acids, hormones, and some other circulating metabolites were associated with risk of dementia, and could serve as potential biomarkers for screening of individuals at risk of dementia and in the development of future therapeutic strategies.

Lipids play critical roles in central nervous system homeostasis, including formation of lipid rafts, maintenance of cell membrane structure, and involvement in signal transduction (Trushina and Mielke, 2014; Han, 2016; Yi et al., 2017). Deficiencies of several lipid classes, such as phospholipid, PC, phosphatidylinositol, sphingolipid, lysophospholipid, SM, and sterols, were found in brain tissue, CSF, and blood in patients with dementia (Oresic et al., 2011; Xu et al., 2012; Trushina and Mielke, 2014; Yi et al., 2017). High levels of PC, SM, and lysoPC were associated with dementia risk in our systematic review (Oresic et al., 2011; Li et al., 2017; Toledo et al., 2017; Varma et al., 2018). Toledo et al. (2017) reported that PC and SM were associated with abnormal CSF Aβ_1−42_ values, supporting the theory that lipid rafts could facilitate the aggregation of Aβ, which may indicate loss of membrane function and neurodegeneration in early stage cognitive dysfunction (Rushworth and Hooper, 2010). Thus, SM- and PC- containing lipids varied with time with the onset of dementia, with higher levels presymptomatically and lower levels post-impairment (Mielke et al., 2010). DHA needs to be supplied by diet, can cross the blood-brain barrier via Mfsd2a (Nguyen et al., 2014), a component of the neuronal cell membrane and anti-inflammatory factor (Cardoso et al., 2016), showed low concentrations in brains of patients with AD (Soderberg et al., 1991), and is associated with cognitive decline (Cardoso et al., 2016). High blood levels of DHA, which are raised by eating fish, were beneficial for cognition and reduced the risk of dementia (van der Lee et al., 2018). Moreover, subclasses of HDL were related to good cognitive performance and lower dementia risk in 11 cohorts (van der Lee et al., 2018). Different HDL subspecies are involved in different functions, including repairing damaged membranes, lipid metabolism, cell signaling, and anti-oxidation, which lead to protective effects on the brain (Shah et al., 2013; van der Lee et al., 2018). Therefore, replication of blood lipid classes might be a more useful endeavor for understanding the basic biology of the preclinical state of dementia in the future.

Several amino acid variations, such as reduced BCAAs (Toledo et al., 2017; Tynkkynen et al., 2018), creatinine (Tynkkynen et al., 2018), and taurine (Chouraki et al., 2017), and higher glutamate (Chouraki et al., 2017), glutamine (van der Lee et al., 2018), and anthranilic acid (Chouraki et al., 2017), are associated with dementia. Alterations in BCAAs were observed in AD-like animal models (Salek et al., 2010; Pan et al., 2016) and patients with AD (Gonzalez-Dominguez et al., 2015; Yi et al., 2017). Essential amino acids could be an energy source whose disruption could indicate nutritional deficiencies and result in lipid membrane changes (Orsitto et al., 2009; Toledo et al., 2017; Tynkkynen et al., 2018). Further, aging caused changes in amino acid metabolism, which was a risk factor for dementia (Kodaira et al., 2017). Notably, as key factors of the glutamine-glutamate/GABA cycle, glutamine, and glutamate were associated with increased risk of dementia (Chouraki et al., 2017; van der Lee et al., 2018). Elevated plasma levels of anthranilic acid and glutamate, and lower levels of taurine were associated with a higher risk of dementia via the tryptophan-kynurenine pathway, which was linked to glutamate excitotoxicity in the pathogenesis of dementia in the Framingham Heart Study (Chouraki et al., 2017). Excess glutamate in brain is likely harmful whereas glutamine is beneficial, since the latter is a non-neuroactive intermediate in the recycling of amino acid neurotransmitters in the brain (Zhou and Danbolt, 2014). All the glutamine in brain is converted from glutamate in astrocytes; therefore, the glutamate and glutamine complex (Glx) represents the integrated metabolism and neurotransmitter functions of glutamate in the brain (Zhou and Danbolt, 2014; Jahng et al., 2016; Huang et al., 2017). Decreased glutamine was found in the brain in animal models of AD (Trushina and Mielke, 2014) and in the serum of patients with AD (Gonzalez-Dominguez et al., 2015; Yi et al., 2017), whereas elevated glutamine was associated with dementia risk in the analyses by van der Lee et al. (2018); this correlation and the underlying mechanisms require further confirmation.

Our systematic review revealed the diversity and complexity of current applications of metabolomics in dementia. Several putative metabolites have been proposed as potential biomarkers for preclinical dementia. However, it is difficult to reach a consistent conclusion, since biomarker validation in different populations is a major challenge.

Several aspects should be taken into consideration. First, differences among articles in study design, populations, cognitive testing, and outcome definitions introduce heterogeneity and affect evaluations and replications in independent populations. Most of these studies used cohort designs and recruited participants from the community, while two analyses included participants selected from memory clinics or prevention trials (Graham et al., 2015; Abdullah et al., 2017). However, nearly all of the relevant studies were based on retrospective analysis of samples collected for other outcomes or samples that were collected “just in case.” Prospective studies designed from conception to look specifically at metabolomics in dementia with appropriate participant recruitment, biological sample collection, processing, and storage are warranted. Moreover, the baseline cognitive status of participants [participants with MCI in three analyses (Oresic et al., 2011; Graham et al., 2015; Toledo et al., 2017)], methods used to identify outcomes, and durations of follow-up were different across all the included studies, and the metabolic profile may be different in different phases of disease (Trushina and Mielke, 2014). Further, age, sex, and race may also contribute to disparities in metabolite profile and prevalence of dementia (Jove et al., 2014; Trushina and Mielke, 2014; Gonzalez-Dominguez et al., 2017; Li et al., 2017), which could result in discrepancies in replication and predictive analyses.

Second, the utilization of diverse metabolomics analytical platforms and different biological samples (plasma or serum) resulted in variations in the data. MS-based methods coupled with variations in separation technique (i.e., gas chromatography [GC], liquid chromatography [LC], capillary electrophoresis [CE], etc.) were applied in all 16 studies, MS and NMR were both included in three articles (Toledo et al., 2017; Tynkkynen et al., 2018; van der Lee et al., 2018) in different cohorts, and only one cohort (the FINRISK 1997 study) used both LC-MS and NMR in serum (Tynkkynen et al., 2018). Eight analyses used untargeted approaches, while eight applied targeted approaches, which could result in differences in metabolite coverage. Each of these platforms has advantages and disadvantages, and none could capture the entire metabolomics map, which results in inconsistencies in the detected metabolites (Kaddurah-Daouk et al., 2008; Xu et al., 2012; Trushina and Mielke, 2014). Eight studies used serum, seven used plasma, and one used both (Tynkkynen et al., 2018). Samples were not collected in the same conditions. In addition, sample collection and storage were not uniform: the sample freeze time and freeze-and-thaw times were different. All these factors could cause inconsistencies and difficulties in the extrapolation of results (Breier et al., 2014). To link these variables directly to the studies in this review, for example, using the same metabolomics platform, Casanova et al. (2016) failed to replicate the metabolite panel that could predict AD found by Mapstone et al. (2014), which might largely be due to the different biological samples used in the two studies (plasma vs. serum).

Third, differences in statistical approach could be a further source of inconsistency. All the studies included in this systematic review aimed to identify individual circulating metabolites or a combination of metabolites related to cognitive decline or incident dementia (MCI or all-cause dementia). Cox (Mielke et al., 2010; Abdullah et al., 2017; Bressler et al., 2017; Chouraki et al., 2017; Tynkkynen et al., 2018; van der Lee et al., 2018) or logistic (Oresic et al., 2011; Mapstone et al., 2014; Casanova et al., 2016; Li et al., 2017) regression models were applied to evaluate the association of metabolites at baseline and incident dementia, while linear models were used in terms of cognitive decline (Casanova et al., 2016; Bressler et al., 2017; Li et al., 2017; Toledo et al., 2017). Three studies used data-driven multivariate methods, such as orthogonal to partial least squares discriminant analysis (OPLS-DA) (Mousavi et al., 2014; Graham et al., 2015) and machine learning (i.e., RF, SVM, etc.; Casanova et al., 2016; Varma et al., 2018). Different data reduction approaches and multiple testing correction were applied in some of the studies, including principal component analysis (PCA) (Tynkkynen et al., 2018), LASSO (Mapstone et al., 2014), Bayesian model-based clustering (Oresic et al., 2011), Bonferroni or false discovery rate (FDR) correction (Mapstone et al., 2014; Casanova et al., 2016; Abdullah et al., 2017; Toledo et al., 2017; Dorninger et al., 2018), and others (Bressler et al., 2017; Chouraki et al., 2017; van der Lee et al., 2018). In addition, pretreatment of metabolite data, including scaling, normalization and transformation, sample size, and covariates in adjusted models were inconsistent across different studies. All of these are critical aspects in data-mining, and could affect the selection of metabolites (Guasch-Ferre et al., 2016; Ruiz-Canela et al., 2017; Yi et al., 2017).

Taken together, there is a need to standardize the workflow for study design, participant selection, sample collection and storage, and experimental and data procedures (Lindon et al., 2005; Yi et al., 2017). Application of combined analytical platforms is an attractive prospect for future metabolomics studies (Xu et al., 2012; Yi et al., 2017). Moreover, it is imperative to conduct studies in larger, well-phenotyped, serial biospecimens, with more genetic background cohorts to enhance the identification of metabolites in the prediction of dementia, especially in stages before MCI.

Several limitations of this systematic review should be acknowledged. First, our search was limited to English, which excluded studies that were published in other languages. However, most articles are published and indexed in English in PubMed, Embase, and the Cochrane Library. Second, we only included blood metabolites measured by high-throughput analytical approaches, which would exclude studies evaluating only one or several metabolites before the development of metabolomics, or conducted in other types of biospecimens related to risk of dementia. However, we aimed at identifying circulating metabolites in metabolomics profiles associated with cognitive decline or progression to dementia, which could be promising biomarkers of preclinical dementia. Third, we were unable to conduct a quantitative meta-analysis, owing to the inconsistency of the results of the included articles.

## Conclusion

Metabolomics is a promising novel approach to understand the pathophysiology of dementia and identify biomarkers for the risk of dementia. This systematic review provides evidence that several metabolites, including lipids (mainly PC, lyso PC, SM, and HDL subfractions), amino acids (BCAAs, glutamate, etc.), and steroids are associated with cognitive performance change and risk of dementia. These findings highlight the translational strength of metabolomics and heterogeneities among current studies. To improve the identification of potential biomarkers of dementia, standardized designs, and experimental protocols for metabolomics studies in larger number of participants, longer follow-up time, and serial blood samples in diverse populations are warranted.

## Author Contributions

XC, MC, and YJ designed this review. YJ, ZZ, and JS searched literature and assessed their quality. YJ wrote the initial manuscript. LJ and WY supervised and provided critical comments on the manuscript. All the authors (YJ, ZZ, JS, YA, KZ, YW, SL, LJ, WY, MC, and XC) read, amend, and discussed the article.

### Conflict of Interest Statement

The authors declare that the research was conducted in the absence of any commercial or financial relationships that could be construed as a potential conflict of interest.
