# Supplementary material for: Metabolomics in the Development and Progression of Dementia: A Systematic Review
Source: Front Neurosci. 2019 Apr 12;13:343. doi: 10.3389/fnins.2019.00343 (PMC6474157; doi:10.3389/fnins.2019.00343)
Supplement: Supplementary file 1 [file Table_1.DOCX]

**Supplementary Table 1. Search Strategy and terms PubMed, Embase, and the Cochrane Library**

| **Database** | **Search-terms** |
| --- | --- |
| PubMed | (“metabolomics” [MeSH Terms] OR “metabolome” [MeSH Terms] OR “metabolomics” [All Fields] OR “metabolome” [All Fields] OR “metabonomics” [All Fields] OR “metabolites” [All Fields] OR “lipidomics” [All Fields] OR “metabolic profil*” [All Fields] OR “metabolic network*” [All Fields])  AND (“Dementia” [MeSH Terms] OR “Alzheimer Disease” [MeSH Terms] OR “Cognitive Dysfunction” [MeSH Terms] OR “Dementia” [All Fields] OR “Alzheimer” [All Fields] OR “Cognitive Dysfunction” [All Fields] OR “Cognitive Decline” [All Fields] OR “Cognitive Impairment” [All Fields])  AND “humans” [MeSH Terms]  AND (“0001/01/01”[PDat] : “2018/10/15”[PDat])  AND English [Language]  NOT systematic review[Publication Type] NOT review[Publication Type] NOT Meta-analysis[Publication Type] NOT case reports[Publication Type] NOT clinical conference[Publication Type] NOT congresses[Publication Type] NOT editorial[Publication Type] NOT addresses[Publication Type] |
| Embase | (“metabolomics”/exp OR “metabolomics” OR “metabolome”/exp OR “metabolome” OR “metabonomics”/exp OR “metabonomics” OR “metabolites”/exp OR “metabolites” OR “lipidomics”/exp OR “lipidomics” OR “metabolic profil*” OR “metabolic network*”)  AND (“dementia”/exp OR “dementia” OR “alzheimer disease”/exp OR “alzheimer” OR “cognitive defect”/exp OR “cognitive defect” OR “cognitive dysfunction” OR “cognitive decline” OR “mild cognitive impairment”/exp OR “cognitive impairment”)  NOT ([systematic review]/lim OR [review]/lim OR [conference abstract]/lim OR [conference paper]/lim OR [conference review]/lim OR [editorial]/lim OR [erratum]/lim OR “nonhuman”/de) NOT [15-10-2018]/sd  AND [english]/lim |
| Cochrane | ((“metabolomics” [MeSH] OR “metabolome” [MeSH] OR “metabolomics” OR “metabolome” OR “metabonomics” OR “metabolite” OR “lipidomics” OR “metabolic profil*”)  AND (“Dementia” [MeSH] OR “Alzheimer Disease” [MeSH] OR “Cognitive Dysfunction” [MeSH] OR “Dementia” OR “Alzheimer” OR “Cognitive Dysfunction” OR “Cognitive Decline” OR “Cognitive Impairment”)  Date added to CENTRAL trials database: ~ to 15/10/2018 |

**Supplementary Table 2. Quality assessment for cohort studies using a modification of the Newcastle–Ottawa Scale**

| Study | Selection | | | | Comparability | Outcome | | Score |
| --- | --- | --- | --- | --- | --- | --- | --- | --- |
| First Author, Year, Journal | Representativeness of the exposed cohort | Selection of the non-exposed cohort | Ascertainment of exposure | Demonstration that outcome of interest was not present at start of study | Control for important factors (adjustment) | Assessment of outcome | Follow-up long enough for outcomes to occur (follow-up duration) |  |
| Mielke, 2010, Neurobiol Aging | 1 | 1 | 1 | 1 | 2 | 1 | 1 | 8 |
| Oresic, 2011, Transl Psychiatry | 0 | 0 | 1 | 1 | 1 | 1 | 0 | 4 |
| Simpson, 2016, J Cereb Blood Flow Metab | 1 | 1 | 1 | 1 | 2 | 1 | 1 | 8 |
| Bressler, 2017, Transl Psychiatry | 1 | 1 | 1 | 0 | 2 | 1 | 1 | 7 |
| Chouraki, 2017, Alzheimers Dement | 1 | 1 | 1 | 1 | 2 | 1 | 1 | 8 |
| Li, 2017, Alzheimers Dement (DADM) | 1 | 1 | 1 | 1 | 2 | 1 | 1 | 8 |
| Toledo, 2017, Alzheimers Dement | 1 | 1 | 1 | 1 | 2 | 1 | 0 | 7 |
| Tynkkynen, 2018, Alzheimers Dement | 1 | 1 | 1 | 1 | 2 | 1 | 1 | 8 |
| van der Lee, 2018, Alzheimers Dement | 1 | 1 | 1 | 1 | 2 | 1 | 1 | 8 |
| Varma, 2018, PloS Med | 1 | 1 | 1 | 1 | 1 | 1 | 0 | 6 |

Note. Scoring criteria were defined as follows: Representativeness of the exposed cohort: 1 point if participants were selected from population-based study; Selection of the non-exposed cohort: 1 point if participants were selected from the same population as the exposure cohort; Ascertainment of exposure: 1 point if description of metabolomics method and metabolites analyzed; Demonstration that outcome of interest was not present at start of study: 1 point if there was a demonstration; Control for important factors (adjustment): 2 point if the analysis was adjusted for most potential confounders, and 1 point if the analysis was only adjusted for age and sex (or age and sex matched); Assessment of outcome: 1 point if exposure was defined by study measurement or medical records; Was follow-up long enough for outcomes to occur ( follow-up duration): 1 point if the follow-up duration was longer than 5 years; points were summed ranged from 0 to 8, studies with summed scores ≤4 were considered to be of low quality.

**Supplementary Table 3.  Quality assessment for case-control studies using a modification of the Newcastle–Ottawa Scale**

| Study | Selection | | | | Comparability | Exposure | | Score |
| --- | --- | --- | --- | --- | --- | --- | --- | --- |
| First Author, Year, Journal | Adequate definition of cases | Representativeness of the cases | Selection of controls | Definition of controls | Control for important factors (adjustment) | Assessment of exposure | Same method of ascertainment for cases and controls |  |
| Mapstone, 2014, Nat Med | 1 | 1 | 1 | 1 | 2 | 1 | 1 | 8 |
| Mousavi, 2014, Dement Geriatr Cogne Disord Extra | 1 | 1 | 1 | 1 | 1 | 1 | 1 | 7 |
| Graham, 2015, PLoS One | 1 | 0 | 0 | 1 | 0 | 1 | 1 | 4 |
| Casanova, 2016, Alzheimers Dement | 1 | 1 | 1 | 1 | 1 | 1 | 1 | 7 |
| Abdullah, 2017, Aging | 1 | 0 | 0 | 1 | 2 | 1 | 1 | 6 |
| Dorninger, 2018, J Alzheimers Dis | 1 | 1 | 1 | 1 | 2 | 1 | 1 | 8 |

Note. Scoring criteria were defined as follows: Adequate definition of cases: 1 point if exposure was defined by study measurement or medical records; Representativeness of the cases: 1 point if participants were selected as consecutive or obviously representative series of cases; Selection of controls: 1 point if participants were selected from community; Definition of controls: 1 point if participants were no history of interested cognitive impairment at both baseline and follow-up; Control for important factors (adjustment): 2 point if the analysis was adjusted for most potential confounders, and 1 point if the analysis was only adjusted for age and sex (or age and sex matched); Assessment of exposure: 1 point if description of metabolomics method and metabolites analyzed; Same method of ascertainment for cases and controls: 1 point if the metabolic profiling approach and protocol was same with cases; points were summed ranged from 0 to 8, studies with summed scores ≤4 were considered to be of low quality.
